# Preoperative virtual reality-guidance for safe gastric endoscopic full-thickness resection with a suitable closure strategy

**DOI:** 10.1055/a-2622-4724

**Published:** 2025-07-09

**Authors:** Noriko Nishiyama, Kaho Nakatani, Ryosuke Kawanishi, Shintaro Fujihara, Bumpei Nishiura, Takayoshi Kishino, Hideki Kobara

**Affiliations:** 1 Departments of Gastroenterology and Neurology, Faculty of Medicine, Kagawa University, Kagawa, Japan; 2 Nishiyama Memorial Medical Corporation, MIRAI Hospital, Kanazawa, Japan; 3 Department of Gastroenterological Surgery, Faculty of Medicine, Kagawa University, Kagawa, Japan

Gastric endoscopic full-thickness resection (gEFTR) is an advanced endoscopic technique for gastric subepithelial tumors (gSETs) 1 . However, unlike surgeons, endoscopists often are not thoroughly familiar with the anatomy outside the gastric wall. Furthermore, during full-thickness resection, this lack of familiarity carries a high risk of damaging large arteries and veins that supply the tumor, leading to the loss of visualization and conversion to open surgery. Therefore, to perform gEFTR safely, it is ideal to assess the surrounding anatomy and feeding vessels preoperatively. Three-dimensional (3D) holograms with virtual reality (VR) and mixed reality technology as surgical navigation support tools 2 3 have been currently introduced in endoscopic biliary procedures 4 . We report the first known case in which VR was used for gEFTR.

A 74-year-old man presented with anterior wall gSET in the upper stomach ( Fig. 1 **a, b** ). 3D images of the stomach and surrounding organs were created preoperatively from contrast-enhanced computed tomography images using Ziostation2 (Ziosoft, Inc., Tokyo, Japan). The images were converted to 3D polygon data using the Holoeyes XR system (Holoeyes Inc., Tokyo, Japan) installed in a head mount display (Meta Quest3; Meta Platforms, Menlo Park, CA, USA) ( Fig. 2 ). The tumor location was confirmed close to the liver, and a feeding artery and vein were identified from the forward and retroflexed views ( Fig. 3 , Fig. 4 **a,b** ). gEFTR ( Video 1 ) was then completed safely, with no bleeding. The diameter of the anticipated full-thickness gastric wall defect was estimated at 2 cm preoperatively ( Fig. 5 **a,b** ), suggesting the closure strategy; inverted closure using two over-the-scope clips was achieved.

**Fig. 1:**
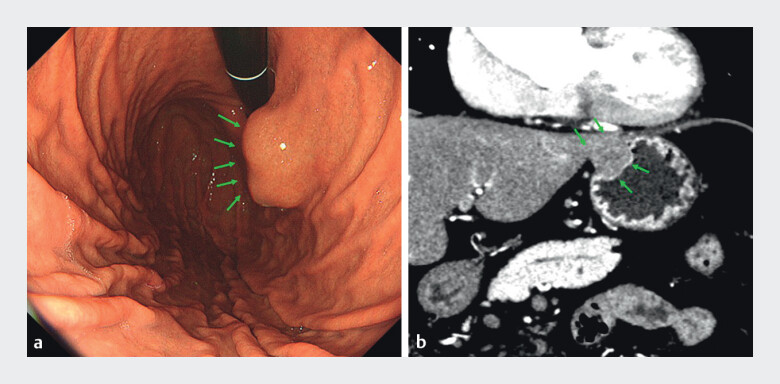
**a** Endoscopic image showing a 2-cm diameter subepithelial tumor (SET) at the anterior wall in the upper stomach. The green arrow indicates the tumor. **b** Contrast-enhanced computed tomography (CT) image showing a 2-cm diameter mass with intra- and extramural growth in the upper body of the stomach. The green arrow indicates the tumor.

**Fig. 2:**
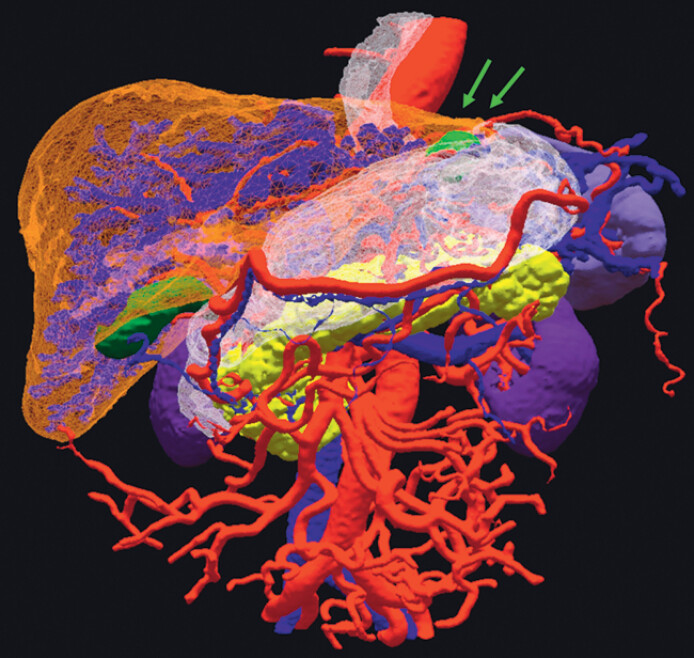
Intraabdominal three-dimensional (3D) holographic image installed in a head mount display using Meta Quest3.

**Fig. 3:**
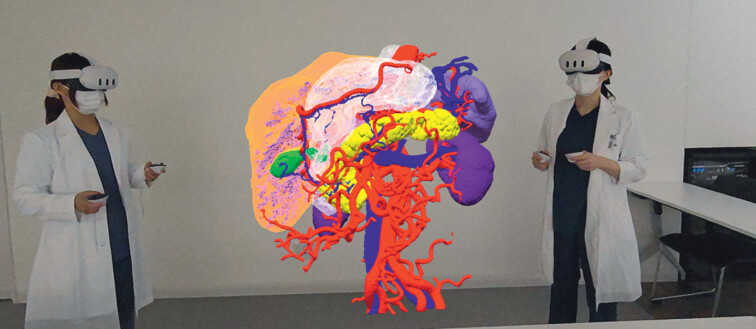
A preoperative multidisciplinary conference was held to review the constructed three-dimensional (3D) hologram.

**Fig. 4:**
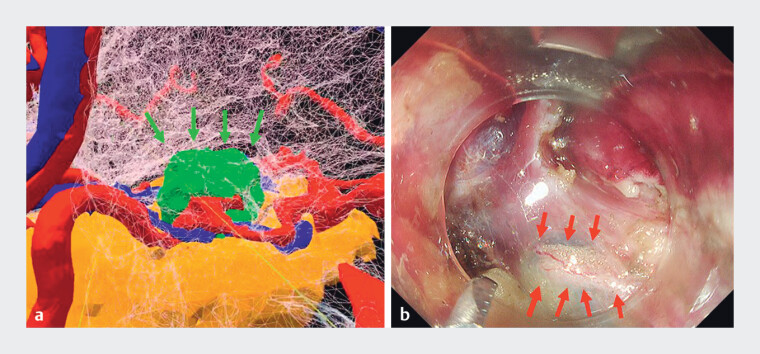
Holographic and endoscopic images. **a** Identifying a tumor-feeding artery and vein from the forward view from three-dimensional (3D) holographic image. **b** In the endoscopic forward image, an artery feeding the tumor is identified, corresponding to the 3D holographic image. The red arrow indicates the artery.

**Fig. 5:**
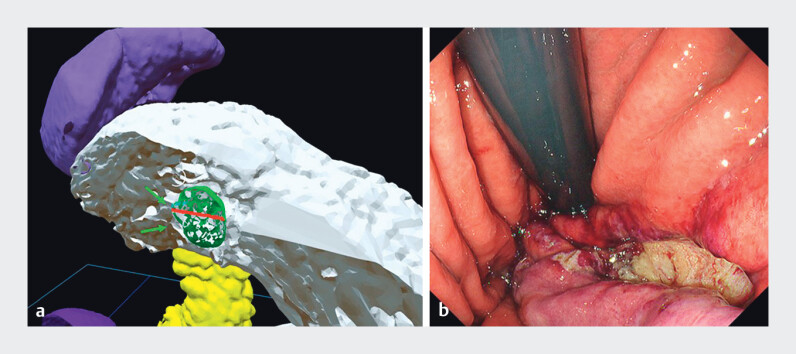
Holographic and endoscopic images. **a** The diameter of the anticipated full-thickness gastric wall defect was estimated at 2 cm preoperatively. The green arrow indicates the tumor. **b** The actual defect after EFTR was approximately 2 cm.

Virtual reality (VR) system using a three-dimensional (3D) hologram for endoscopic full-thickness resection (EFTR).Video 1

Reviewing VR images preoperatively enabled the endoscopist to visualize the anatomy surrounding the tumor and plan a suitable closure strategy.

VR systems using 3D holograms could be useful for safe and reliable gEFTR, with greater operator confidence.

Endoscopy_UCTN_Code_TTT_1AO_2AN

## Notes

Endoscopy E-Videos https://eref.thieme.de/e-videos
*E-Videos* is an open access online section of the journal *Endoscopy* , reporting on interesting cases and new techniques in gastroenterological endoscopy. All papers include a high-quality video and are published with a Creative Commons CC-BY license. Endoscopy E-Videos qualify for HINARI discounts and waivers and eligibility is automatically checked during the submission process. We grant 100% waivers to articles whose corresponding authors are based in Group A countries and 50% waivers to those who are based in Group B countries as classified by Research4Life (see: https://www.research4life.org/access/eligibility/ ). This section has its own submission website at https://mc.manuscriptcentral.com/e-videos .
